# Sarcoidosis-like reaction induced by immune checkpoint inhibitor in a patient with hepatocellular carcinoma: a case report

**DOI:** 10.3389/fimmu.2023.1150128

**Published:** 2023-09-13

**Authors:** Alberto Torres-Zurita, Lucía Vázquez-Montero, Laura Gallego-López, María Dolores Mediano-Rambla, Luis de la Cruz-Merino

**Affiliations:** ^1^ Department of Clinical Oncology, Virgen Macarena University Hospital, Seville, Spain; ^2^ Department of Internal Medicine, Virgen Macarena University Hospital, Seville, Spain

**Keywords:** hepatocellular carcinoma, immune-checkpoint inhibitor, nivolumab plus ipilimumab combination therapy, immune related adverse event (irAE), sarcoidosis

## Abstract

Nowadays, immune checkpoint inhibitors (ICI) have become the cornerstone of treatment for many tumors, either as monotherapy or in combination with other therapies. However, these drugs are associated with several new side effects that need early detection. We present the case of a 41-year-old male patient who has been diagnosed with advanced hepatocellular carcinoma (HCC) with metastatic retroperitoneal lymph nodes and a subdiaphragmatic metastatic lesion, undergoing second-line treatment with a combination of nivolumab and ipilimumab. After completing four cycles, the patient was admitted to the hospital due to intermittent fever and profuse sweating. A CT scan showed multiple pathologically enlarged lymph nodes in several locations, raising suspicion of disease progression. The patient’s clinical progress was favorable after symptomatic treatment (antipyretics) and was discharged one week after admission. Several days later, the patient complained about painful bilateral ocular redness and was diagnosed with bilateral anterior uveitis. Further blood tests showed elevated angiotensin-converting enzyme (ACE) levels of 67 U/L (normal range: 8 – 52) and decreasing alpha-fetoprotein (AFP) levels of 698 ng/mL (previously 1210 ng/mL), indicative of non-progression of the oncological disease. Finally, an excisional biopsy confirmed the presence of non-necrotizing granulomatous lymphadenitis, leading to the diagnosis of sarcoidosis-like reaction (SLR) induced by immunotherapy as the etiology of the polyadenopathy syndrome. SLR, although uncommon, is an adverse effect of ICI treatment resulting from immune system dysregulation, which can mimic disease progression. It is crucial to be aware of this adverse event and to understand the optimal management approach.

## Introduction

The advent of immunotherapy has revolutionized the field of cancer treatment, transforming the trajectory of several tumor types. Notably, humanized monoclonal antibodies such as nivolumab and ipilimumab have emerged as prime examples. These antibodies target the programmed death receptor (PD-1) and cytotoxic T lymphocyte antigen-4 (CTLA-4) respectively, both of which are located on the cell membrane of T lymphocytes.

By exerting their therapeutic effects, these drugs have successfully intercepted one of the cancer cell’s evasion mechanisms: immune response suppression. As a result, they have demonstrated clinical efficacy in malignancies such as melanoma, kidney cancer, and lung cancer. In the case of hepatocellular carcinoma (HCC), the combination of nivolumab and ipilimumab has shown significant activity, leading to its approval for this patient population based on the findings of the CheckMate 040 trial (1).

Immunotherapy has introduced new immune-related adverse events and clinical management challenges, including pseudoprogression and hyperprogression. These circumstances can sometimes be mistaken for immunotherapy-associated side effects, such as sarcoidosis-like reactions (SLRs) (2). To the best of our knowledge, we present the first published clinical case of SLR in a patient undergoing treatment with nivolumab plus ipilimumab for advanced HCC.

## Case report

We present the case of a 41-year-old male patient with an unremarkable medical history who was diagnosed with stage C HCC according to the Barcelona Clinic Liver Cancer classification in October 2020. The carcinoma was located in a non-cirrhotic liver with metastatic retroperitoneal lymph nodes.

In January 2021, treatment with sorafenib 400 mg twice a day was started. However, after nine months, in October 2021, sorafenib was discontinued due to disease progression, characterized by the emergence of a new subdiaphragmatic metastatic lesion and by an increase in alpha-fetoprotein (AFP) levels to 1210 ng/mL (normal range: 0.1 – 10). Subsequently, second-line treatment with nivolumab 1 mg/kg in combination with ipilimumab 3 mg/kg was started based on the findings of the CheckMate 040 trial. The patient received four cycles of this regimen until January 2022.

One week after completing the fourth cycle, the patient was admitted to the hospital due to intermittent fever and profuse sweating. A chest and abdominal CT scan performed during the admission showed multiple pathologically enlarged lymph nodes in several locations (cervical, bilateral hilar, axillary, and inguinal) and hepatosplenomegaly, but it also showed stability in the size of the retroperitoneal lymph nodes and the left subdiaphragmatic lesion. This was further confirmed by positron emission tomography (PET-CT). Consequently, an excisional biopsy of an inguinal lymph node was performed to obtain a histological diagnosis. Considering the patient’s favorable clinical condition and the absence of a definitive diagnosis, it was decided to closely monitor the patient and administer symptomatic treatment with antipyretics. After one week, the fever subsided, and the patient was discharged from the hospital.

A week later, during a follow-up visit at our clinic, the patient complained about bilateral ocular redness accompanied by pain. Ophthalmology consultation lead to the diagnosis of bilateral anterior uveitis. After starting corticosteroids in eye drops, ocular symptoms disappeared in the following days. Given the distribution of the newly appeared lymph nodes, the stability of the previously known disease, and the anterior uveitis, we suspected that we were dealing with a SLR due to immune checkpoint inhibitors. Consequently, a comprehensive blood test, including autoimmunity markers and angiotensin-converting enzyme (ACE) levels, was requested while awaiting the results of the inguinal node biopsy. Given the possibility of disease progression as an alternative diagnosis, repeat alpha-fetoprotein levels were also requested. The immunotherapy was not resumed at this point.

**Figure 1:**
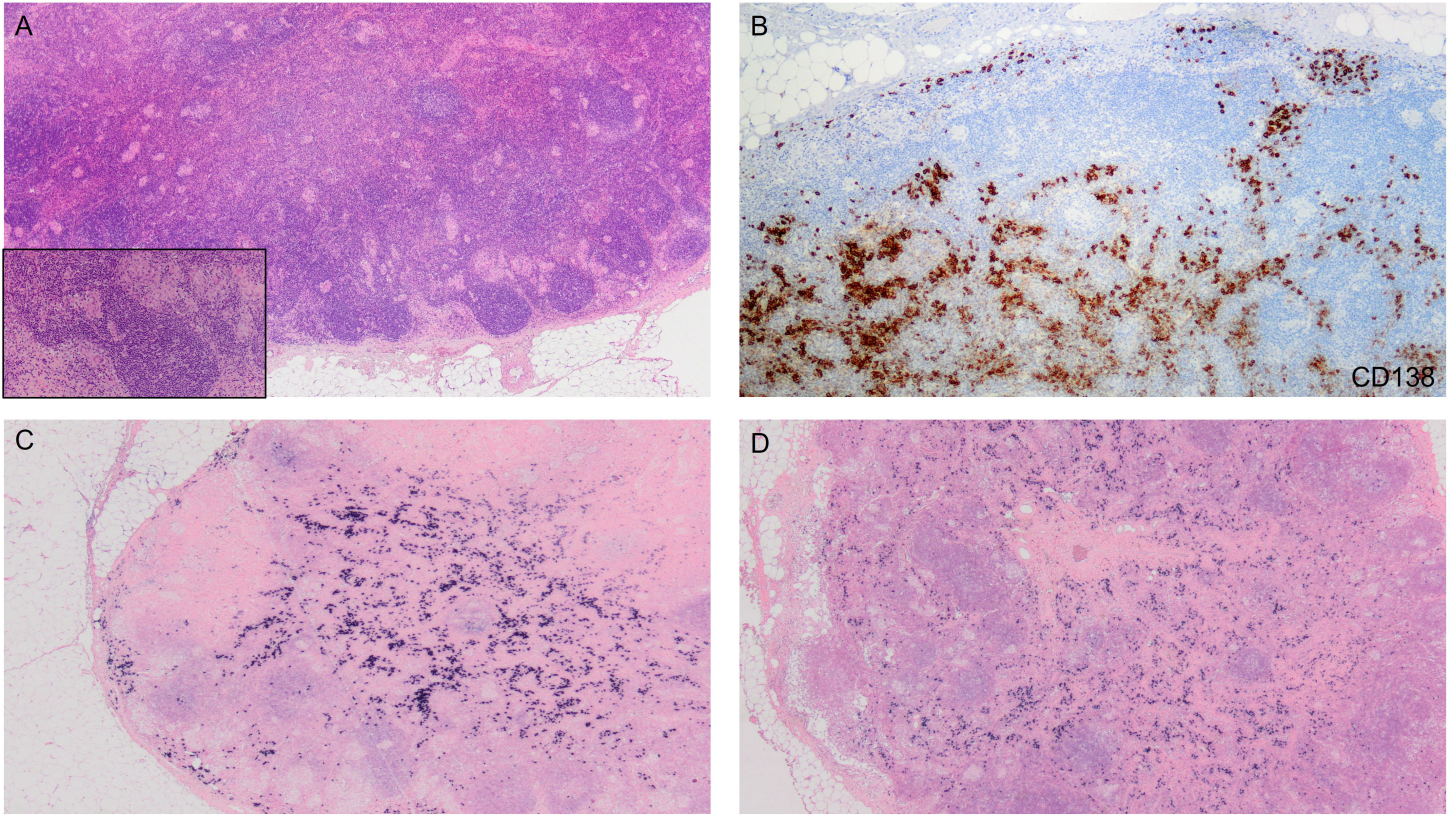
**(A)** Lymph node with the presence of non-epithelioid granulomas adjacent to peripheral primary lymphoid follicles. At higher magnification, confluent granulomas are better visualized, along with the presence of giant cells. (H&E; 2x and 10x). **(B)** Proliferation of plasma cells showing positive immunoreactivity to CD138. (CD138; 4x). **(C, D)** No restriction of kappa or lambda light chains observed. (Kappa and lambda; 2x).

Eventually, the biopsy results showed non-necrotizing granulomatous lymphadenitis (**Figure 1**). The blood test revealed increased ACE levels of 67 U/L (normal range: 8 – 52), decreasing AFP levels of 698 ng/mL (previously 1210 ng/mL), and negative results for autoimmunity markers. Together with the patient’s symptoms, these findings were consistent with SLR following ICI.

Taking into account the disease control achieved with the treatment and the relatively mild symptoms experienced by the patient, it was decided to continue with maintenance nivolumab. After 3 months, a follow-up CT scan was performed, which showed the disappearance of the previously identified lymph nodes observed in the January 2022 CT scan. Furthermore, stability was observed in the retroperitoneal lymph nodes and the left subdiaphragmatic lesion. Consequently, the disease was classified as stable, and the treatment was continued. Currently, the patient remains on nivolumab and has not experienced any new episodes of SLR or disease progression.

## Discussion

Sarcoidosis is a multisystemic granulomatous disease of uncertain etiology characterized by an aberrant immune response mediated by CD4 T lymphocytes. Upon interaction with antigen-presenting cells, these lymphocytes differentiate into helper T lymphocytes type 1 (Th1) and begin to secrete interleukin 2 (IL-2) and interferon gamma (IFN-γ), thereby increasing the production of tumor necrosis factor alpha (TNF-α) by macrophages. Cytokine activation results in the development of organized clusters of epithelioid histiocytes and macrophages surrounded by giant multinucleated cells and lymphocytes (non-necrotizing granulomas). Additionally, helper T lymphocytes type 17 (Th17) have also been involved in the pathogenesis of sarcoidosis (3).

Clinical manifestations vary depending on the affected body part, with pulmonary involvement, mediastinal lymphadenopathy, and cutaneous manifestations being the most characteristic ones. Common associated symptoms include fatigue, fever, night sweats, and weight loss. Extrapulmonary disease can be widespread and affect several organs, such as the eyes (anterior uveitis), the nervous, cardiac, or renal systems and cause hepatosplenomegaly (3).

SLR as an adverse effect of immunotherapy is uncommon but increasingly reported due to the rising use of immune checkpoint inhibitors (ICIs). CTLA-4 blockade has been observed to elevate levels of Th17 lymphocytes in the bloodstream, leading to the increased production of proinflammatory cytokines such as interleukin 17 (IL-17) and TNF-α (4). Furthermore, the PD-1/PD-L1 pathway is associated with the balance of T helper lymphocytes/Th17 lymphocytes, and its blockade enhances Th17 lymphocyte activity and IL-17 expression (5). The blockade of the PD-1/PD-L1 pathway can also activate the mTOR pathway, which is involved in the spontaneous formation of granulomas (6).

The first case-control study of patients with SLR treated with immunotherapy reported 28 cases, mostly in melanoma patients (2). The majority of SLRs were mild to moderate (92.9% grade I-II). Symptoms, especially cough and dyspnea, were present in 53.6% of patients, with radiological findings predominantly in the mediastinal lymph nodes and lung parenchyma, while extrathoracic involvement was less frequent. Elevated ACE levels were observed in 40% of patients. This adverse event was more common when administering the combination of anti-PD1 and anti-CTLA4 therapy (46.4% with nivolumab and ipilimumab combination). The study also showed that patients with SLR had a longer survival. However, due to the heterogeneous population and retrospective nature of the study, drawing conclusions at this stage would be premature. Management of this adverse event varies based on severity, with systemic corticosteroids required in 17.9% of patients in this study, and up to 44% in other series (7). No patients needed immunosuppressive therapy for sarcoidosis management. Immunotherapy was temporarily interrupted in 10.7% of patients and permanently discontinued in 67.9% of patients, with 12 cases attributed to sarcoidosis. The use of corticosteroids should be considered in patients with SLR related to immunotherapy, depending on clinical severity, although it may reduce the efficacy of immunotherapy.

One of the main challenges in patients treated with immunotherapy with SLR is the ability of SLR to mimic disease progression, particularly due to lymph node involvement. In the context of immunotherapy, pseudoprogression and hyperprogression must also be considered. Essential clues in our case included the patient’s good performance status, the abnormal distribution of lymph nodes compared to the previously known disease, the decrease in alpha-fetoprotein levels alongside an increase in ACE levels, and the clinical suspicion of SLR, which was further supported by the presence of typical non-necrotizing granulomas observed in the lymph node histopathological study.

The most notable aspect of our clinical case is the atypical presentation of this SLR due to immunotherapy, with predominantly extrapulmonary involvement (polyadenopathy syndrome, anterior uveitis and hepatosplenomegaly), emphasizing the importance of understanding the full clinical spectrum of this adverse event, which could potentially lead to the discontinuation of oncological treatment due to confusion with disease progression. It is also important to note that this adverse event can arise in various tumor types, including HCC. Our case represents the first clinical report in literature of an advanced HCC patient presenting with SLR induced by ICI.

## Conclusion

SLRs due to immunotherapy are infrequent side effects; however, it is imperative to remain vigilant regarding their occurrence as they may mimic tumor recurrence or progression, leading to treatment discontinuation. Therefore, it is crucial to consider this adverse event and, if suspected, perform a histological study for definitive confirmation.

## Data availability statement

The original contributions presented in the study are included in the article/supplementary material. Further inquiries can be directed to the corresponding author.

## Ethics statement

Written informed consent was obtained from the individual(s) for the publication of any potentially identifiable images or data included in this article. Written informed consent was obtained from the participant/patient(s) for the publication of this case report.

## Author contributions

AT-Z, LV-M and LG-L wrote the first draft of the manuscript. AT-Z did the bibliography review. MM-R and LC-M corrected the latest version of the manuscript. All authors contributed to the article and approved the submitted version.
